# Identifying Tumefactive Demyelination on Synthetic MRI Myelin Maps

**DOI:** 10.1155/crra/8787707

**Published:** 2025-06-02

**Authors:** Brandon Simons, Rebecca Li, Tushar Chandra, Laura Hayes

**Affiliations:** ^1^Department of Radiology, University of Central Florida College of Medicine, Orlando, Florida, USA; ^2^Department of Pediatric Radiology, Nemours Children's Hospital, Orlando, Florida, USA

## Abstract

Tumefactive demyelinating lesions and brain neoplasms often present as a diagnostic challenge due to overlapping radiographic features among conventional imaging modalities ultimately resulting in uncertainty if a biopsy is warranted to establish a definitive diagnosis. Synthetic MRI (SyMRI) is a novel imaging technique providing myelin maps to aid with diagnosis, yet its use in pediatric patients remains largely unexplored. Therein, we report a pediatric case utilizing SyMRI to assist in differentiating tumefactive demyelination from a recurrent glioma. This 16-year-old female with a history of ganglioglioma, presented with sudden left-sided weakness. The initial MRI suggested either a glial neoplasm or a demyelinating lesion, prompting consideration of a biopsy. SyMRI revealed a unique “rim of decreased myelination,” challenging the initial diagnosis. Within 1 week from admission, the patient's symptoms resolved without recurrence. Immunotherapy resolved the demyelinating lesion, supporting the initial SyMRI finding. The case demonstrates the potential of SyMRI in pediatric neuroradiology, highlighting a distinct “rim of demyelination” and its comparison to gliomas aiding in the diagnostic process.

## 1. Introduction

In 1979, tumefactive demyelination was first described by van der Velden et al. in a patient with confirmed multiple sclerosis [1]. Tumefactive demyelination often presents as a solitary demyelinating lesion greater than 2 cm on T2-wieghted MRI. These lesions are most often seen in multiple sclerosis but are also associated with neuromyelitis optica spectrum disorder (NMOSD), acute disseminated encephalomyelitis (ADEM), and others [2]. Current methods of diagnosis for tumefactive demyelinating lesions are magnetic resonance imaging (MRI), positron emission tomography (PET), and cerebral spinal fluid (CSF) analysis [3]. Tumefactive demyelinating lesions and aggressive CNS tumors share similar radiographical and clinical presentations, often resulting in invasive biopsy due to diagnostic uncertainty. Therefore, additional imaging techniques are warranted to further support or reject a diagnosis of tumefactive demyelinating lesion.

Synthetic MRI (SyMRI) is a relatively new imaging technique that utilizes parametric tissue maps based on measurements of magnetic properties in the tissue to generate synthetic (simulated) images [4]. Automatic segmentations and volume measurements of tissues such as white matter, gray matter, cerebrospinal fluid, and myelin are a unique feature of SyMRI, making it useful for tracking disease progression or providing more accurate characterizations of tissue compared to conventional MRI [5]. Currently, SyMRI is more often employed in the context of neurologic conditions in adults, with only a few studies demonstrating its utility in pediatric patients [6]. Its application in pediatric disease has not been fully explored, although it holds potential given the ability of SyMRI to generate multiple contrast-weighted images from a single acquisition, reducing scan time and increasing patient comfort [6]. No health risks are associated with SyMRI besides an additional 5 min in the scanner [6].

Here, we present a case where SyMRI was helpful in suggesting possible tumefactive demyelination rather than recurrent neoplasm in a child with a history of brain tumor.

## 2. Case Presentation

A 16-year-old patient with a past medical history of a left temporoparietal ganglioglioma, status post–gross total resection via craniotomy 8 years ago with resultant seizures, presented to the emergency department with sudden onset left-sided weakness. Six days prior, the patient experienced left lower extremity weakness described as a “heaviness” worse in the distal portion of the extremity with difficulty ambulating. Two days later, the patient also experienced left upper extremity weakness. The patient has a history of generalized epilepsy for the past 8 years, but the seizures are now well controlled with levetiracetam 500 mg daily. Her last seizure was 20 months prior. The patient takes ibuprofen two to three times a week for headaches and rizatriptan for migraines. She denied recent sensory changes, visual disturbances, fever, neck pain or stiffness, changes in mental status, or any other complaints.

On presentation at the emergency department, vital signs were within normal limits, and the mental status was alert and oriented. Neurological examination revealed giveaway weakness throughout the left side. Weakness (3/5) and decreased range of motion with plantar and dorsiflexion of the left foot were noted. The patient was unable to move the left foot digits 2–5 and could only slightly move the large left toe. Also, the patient had an impaired gait and a sluggish pupillary reflex. No other abnormalities were detected on physical examination. The erythrocyte sedimentation rate was slightly elevated at 37 mm/h (< 20 mm/h), and the remaining routine laboratory tests were within normal limits.

## 3. Methods

SyMRI produced by SyntheticMR AB (SE-582 23 Linköping, Sweden) was added to standard MRI protocols in this case to aid in diagnosis.

### 3.1. Imaging Findings

**Figure 1:**
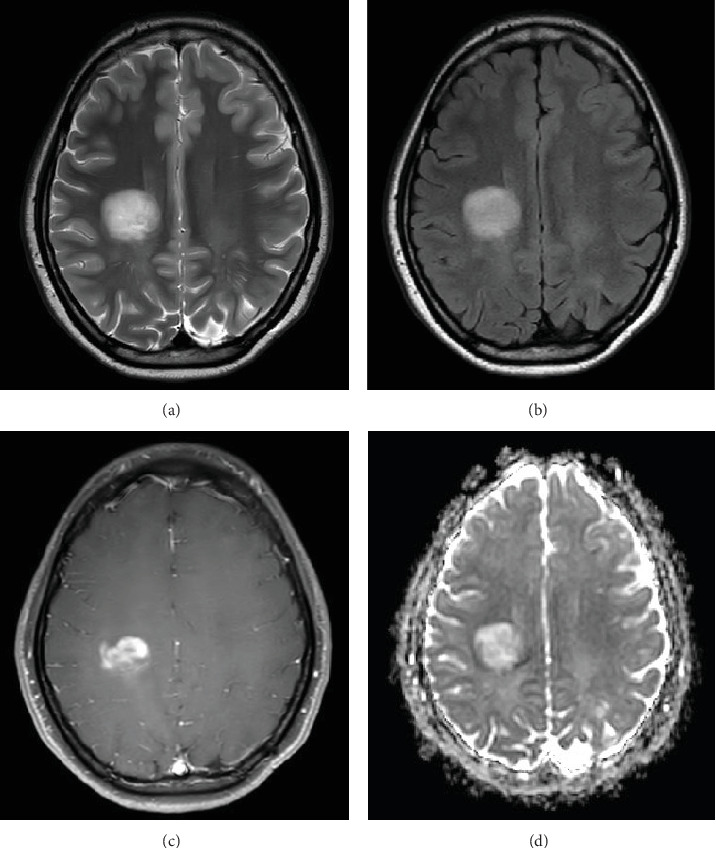
Axial images from brain MRI (on the day of admission) demonstrating a focal, mildly heterogeneous, 2.9 × 2.4 cm hyperintense area of signal abnormality on (a) T2 TSE and (b) T2 FLAIR. On the postcontrast T1-weighted image (c), there is a patchy, heterogeneous enhancement within the lesion. DWI ADC map (d) reveals areas of low ADC values within and around the periphery of this lesion.

**Figure 2:**
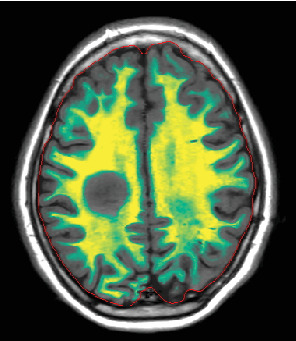
Axial image from synthetic MRI myelin maps (on the day of admission) reveals a uniform “ring of decreased myelination” surrounding the lesion.

Since the ganglioglioma resection 8 years ago, the patient had not received follow-up imaging surveillance. An MRI scan of the brain was performed on the day of admission which demonstrated multiple focal intraparenchymal lesions with T2/FLAIR hyperintensity, the largest of which was a 2.9 × 2.4 cm enhancing lesion in the right centrum semiovale (Figure 1). MR spectroscopy displayed a tall, sharp choline peak with a decreased *N*-acetylaspartate (NAA) and an elevation of lipid lactate. DWI/ADC imaging demonstrated areas of decreased ADC values, indicating areas of decreased diffusivity (Figure 1). Conventional sequences (T2 and FLAIR), DWI/ADC maps, and MR spectroscopy results were consistent with either a high-grade neoplasm or demyelinating lesion. Given the patient's oncologic history, many of the patient's caregivers initially believed the scan revealed recurrent tumor and began preparations for biopsy. However, the presence of multiple small lesions was confusing, and a tumefactive demyelination was considered in the differential. Furthermore, the myelin map derived from SyMRI revealed unique findings. The technique revealed a “rim of decreased myelination,” characterized as a thin, concentric, and uniform decrease in myelin volume surrounding the area of abnormal signal and enhancement (Figure 2). This concentric, well-defined rim was identified to be different from the irregular, ill-defined edge of decreased myelination seen with infiltrating, high-grade gliomas. The presence of this unique myelination pattern allowed the team to reconsider her initial diagnosis of tumor recurrence.

On the first day after admission, the patient's weakness improved. Lumbar puncture revealed clear, colorless cerebrospinal fluid with normal glucose, protein, and cell count. Not enough cerebrospinal fluid was collected to evaluate for oligoclonal bands.

Due to the patient's rapid clinical improvement and stability, she was discharged with repeat MRI brain and lumbar puncture for oligoclonal bands scheduled in 3 weeks to determine if the studies were more consistent with demyelination or tumor.

**Figure 3:**
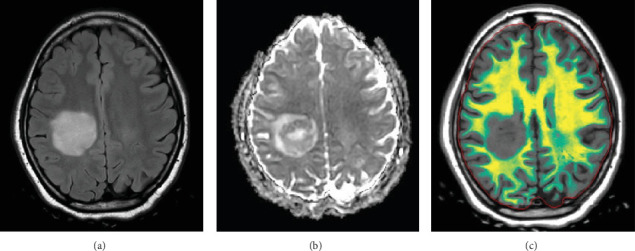
Axial images from brain MRI (3 weeks after admission) demonstrating a hyperintense lesion on (a) T2 FLAIR in the right centrum semiovale and corona radiata measuring 3.7 × 3.7 cm. The lesion demonstrates decreased ADC values (b) in keeping with reduced diffusivity. Synthetic MRI myelin map (c) demonstrates a “ring of decreased myelination” around the lesion.

Follow-up MRI brain demonstrated a significant interval enlargement in size of the lesion, measuring 3.7 × 3.7 cm in the axial plane compared to 2.9 × 2.4 cm on the prior study (Figure 3). The same “ring of decreased myelination” was once again seen (Figure 3). Three oligoclonal bands were observed in the cerebrospinal fluid, which was below the diagnostic criteria for multiple sclerosis of four or more oligoclonal bands. Cytology of the cerebrospinal fluid revealed no malignant cells. Myelin oligodendrocyte glycoprotein antibodies, aquaporin-4 antibodies, ANA comprehensive panel, antiphospholipid panel, anticardiolipin antibodies, B-2-G, and ANCA profile were all negative.

The patient's symptoms completely resolved within 1 week of discharge. At 1-month postdischarge, she denied any new symptoms concerning demyelinating relapse and was diagnosed with a demyelination disease. Six hundred milligrams of IV ocrelizumab every 6 months was started. Treatment with immunotherapy resolved the lesion.

## 4. Discussion

Tumefactive demyelinating lesions are characterized on MRI as large white matter lesions with complete or open-ring enhancement, a T2 hypointense rim, and peripheral diffusion restriction [3]. They may easily be misdiagnosed as neoplastic lesions, as these radiological features can be diverse and enhancement patterns can be variable [3].

Recently, the central vein sign (CVS) has been proposed as an additional imaging marker within the McDonald criteria for diagnosing multiple sclerosis [7]. The CVS is a specific biomarker indicative of perivenous inflammatory demyelination. The CVS appears as a centrally located small (< 2 mm) hypointense line or dot visible in at least two perpendicular MRI planes on susceptibility-weighted or T2-weighted images [2]. Studies suggest that the CVS is present in approximately half of tumefactive demyelinating lesions [8]. However, the CVS was absent in this case adding to the diagnostic uncertainty.

The patient's initial brain MRI was consistent with either a neoplasm or a demyelinating lesion. In this patient with a history of brain tumor, concern for tumor recurrence increased the likelihood of an aggressive neoplasm, and the patient was considered for biopsy.

A 2D SyMRI sequence was added into standard MRI acquisition protocols for patients with possible/confirmed CNS tumors who undergo MRI as part of standard of care. The synthetic images presented unique findings regarding the patient's lesion.

The myelin map derived from SyMRI revealed a “rim of decreased myelination,” which challenged the initial diagnosis of a tumor. The “rim of decreased myelination” was characterized as a thin, consistent, and uniform decrease in myelin volume around the lesion.

**Figure 4:**
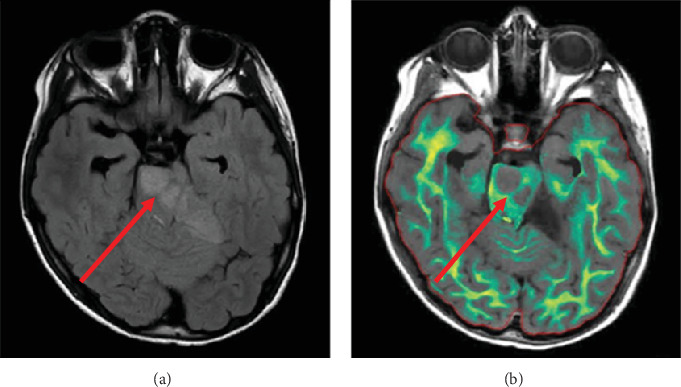
Brain MRI with myelin maps of high-grade glioma. Axial brain MRI demonstrating a high-grade diffuse midline glioma (red arrow) located in the pons on (a) FLAIR. (b) Myelin maps reveal an ill-defined, irregular margin of decreased demyelination.

The patient's myelin maps were compared to SyMRI images of confirmed high-grade gliomas. Myelin maps of a high-grade glioma demonstrated an ill-defined, irregular margin of decreased demyelination (Figure 4).

Furthermore, the “rim of decreased myelination” may also be related to the pathogenesis of tumefactive demyelinating lesions. Although the literature is unclear on the pathogenesis, one study focusing on the differential gene expression profiles between glial cells derived from tumefactive demyelinating lesions and glioma revealed that there is an upregulation of myelin-related genes in glial cells derived from tumefactive demyelinating lesions [9]. This finding may elucidate the role of glial cells in the remyelination process in tumefactive demyelinating lesions.

In this case, the “rim of decreased myelination” helped to provide earlier suspicion of tumefactive demyelinating lesion over tumor. This finding reveals the utility of SyMRI in providing additional information that can aid in clinical decision-making, especially in the absence of a CVS. Ultimately, given the lesion's location, a biopsy was deferred due to the risk of permanent disability and the possibility of tumefactive demyelination based on imaging. Ultimately, the patient's subsequent diagnosis of multiple sclerosis along with the resolution of the demyelinating lesion with immunotherapy provided greater validation of the initial “rim of decreased myelination” sign seen on the myelin map.

## 5. Conclusion

The significance of the “rim of decreased myelination” must be further explored to establish conclusions about the specificity or predictivity of this sign for tumefactive demyelinating lesions. This case underscores the importance of this unique finding on the myelin map, which may in the future provide earlier differentiation of an acute demyelinating lesion from a high-grade neoplasm and may help avoid risky invasive procedures in the future.

## Data Availability

The data that support the findings of this study are available from the corresponding author upon reasonable request.
